# Notch and Wnt Dysregulation and Its Relevance for Breast Cancer and Tumor Initiation

**DOI:** 10.3390/biomedicines6040101

**Published:** 2018-11-01

**Authors:** Eike-Benjamin Braune, Anita Seshire, Urban Lendahl

**Affiliations:** 1 Department of Cell and Molecular Biology, Karolinska Institute, SE-171 77 Stockholm, Sweden; eike-benjamin.braune@ki.se; 2 Merck KGaA, Biopharma, Global R&D, Translational Innovation Platform Oncology, Frankfurter Str. 250, D-64293 Darmstadt, Germany; anita.seshire@merckgroup.com

**Keywords:** breast cancer, cancer stem cell, tumor therapy, Notch signaling, Wnt signaling

## Abstract

Breast cancer is the second leading cause of cancer deaths among women in the world. Treatment has been improved and, in combination with early detection, this has resulted in reduced mortality rates. Further improvement in therapy development is however warranted. This will be particularly important for certain sub-classes of breast cancer, such as triple-negative breast cancer, where currently no specific therapies are available. An important therapy development focus emerges from the notion that dysregulation of two major signaling pathways, Notch and Wnt signaling, are major drivers for breast cancer development. In this review, we discuss recent insights into the Notch and Wnt signaling pathways and into how they act synergistically both in normal development and cancer. We also discuss how dysregulation of the two pathways contributes to breast cancer and strategies to develop novel breast cancer therapies starting from a Notch and Wnt dysregulation perspective.

## 1. Introduction

Each year, more than a quarter of a million new breast cancer patients are diagnosed in the US alone, making breast cancer an important medical and societal problem. While treatment has improved, both because of more widespread scanning for early stages of breast cancer by mammography and development of new treatment modalities (see below), still more than 40,000 patients in the US succumb to breast cancer every year. Breast cancer is a heterogeneous disease but thanks to improved immune-histological analysis and a better molecular understanding of the tumor cells, we have begun to classify breast cancer into a number of subgroups. This is important not only to understand the heterogeneity of this cancer form but also for the choice of appropriate therapy. Thus, a major classification is based on the presence or absence of estrogen receptor alpha (ERα), the progesterone receptor (PR) and the human epidermal receptor 2 (Her2). Her2-positive breast tumors can be treated by drugs directed at Her2 (such as trastuzumab), whereas endocrine therapy (such as tamoxifen and raloxifen) is used in treatment of the ERα and PR forms of breast cancer. There is also an important subclass of breast tumors that are devoid of ERα, PR and Her2 expression, referred to as triple-negative breast cancer (TNBC). TNBC accounts for at least 10–15% of all breast cancers and poses a huge medical problem, as there are currently no targeted drugs having been approved for this aggressive form of breast cancer. Analysis of gene expression at the transcriptional level has produced further insights into the nature of tumor heterogeneity. Transcriptional profiling reveals that breast cancers can be stratified into five major subgroups: luminal A, luminal B, Her2-enriched, claudin-low and basal-like [1,2]. These molecular subtypes relate to the immunohistochemistry-based subgroups: the luminal A and B classes are both positive for ERα, Her2-enriched are positive for Her2 whereas the basal-like subgroup overlaps with TNBC. Despite the progress in classification, our understanding of the heterogeneity of breast cancer is still limited, and in particular the TNBC category is likely to be heterogeneous, as a number of TNBC subtypes have recently been identified [3].

A major cause of death from breast cancer results from dissemination of cells that migrate away from the original tumor to metastasize to other parts of the body, which occurs in up to 30% of women diagnosed with breast cancer. Spreading of cells occurs via the lymphatic system, and bone is the most common metastatic site (approximately 70% of all metastases), followed by lungs, regional lymph nodes, blood supply, brain and liver. Cells that are disseminated from the original tumor are more therapy resistant (for review see [4]). Understanding the basis for this acquired therapy resistance as well as the relationship between tumor cells and the tissue microenvironment at the metastatic sites are currently highly active research areas.

To establish which cells in the breast tumors that are drivers of tumor growth is vital, as they are interesting candidates for tumor therapy. The improved understanding of the cellular composition and heterogeneity of breast tumors has allowed this question to be more systematically addressed. While it was traditionally considered that most cells in a tumor could spawn new tumors, the concept that only a small subset of cells in the tumor—the cancer stem cells (CSCs)—are endowed with this potential has gradually gained ground. The CSC concept originates from pioneering studies of leukemia by John Dick and colleagues, who in the 1990s showed that only a very small number of leukemic cells were sufficient to produce a new tumor in transplantation experiments in mice [5]. Al-Hajj et al. (2003) [6] showed that the situation was similar in breast cancer: a small population of cells were identified that could generate mammary tumors in mice upon transplantation. Originally, breast cancer stem cells (BCSCs) were defined by their expression of Cluster of Differentiation (CD) CD44 and CD24 (CD44^+^/CD24^−/low^) [6], and subsequent studies have provided more detailed insights into the molecular nature of the BCSCs. Expression of the epithelial cell marker EpCam has been associated with higher tumorigenic potential [7], and sorting for aldehyde dehydrogenase activity has identified an additional BCSC population, which partly overlaps with CD44^+^/CD24^−/low^ BCSCs [8,9]. Other biomarkers, such as Aldehyde Dehydrogenase 1 (ALDH1), CD133 and CD49f have also been associated with BCSCs, and higher expression levels of these markers are found in TNBC [9], which may be linked to the aggressive nature of TNBC tumors. While the BCSC concept is becoming more widely accepted, the origin of BCSCs is still a matter of debate. Dedifferentiation from more mature cell types as well as an origin from mammary stem cells (MASCs) have been proposed. Furthermore, epithelial-to-mesenchymal transition (EMT) has been shown to generate cells with more stem cell-like properties in the mammary system [10,11]. To further complicate matters, the nature of the cell lineage tree for the principal mammary cell types, myoepithelial and luminal cells, is not well understood, and evidence for a unipotent progenitor cell as well as the existence of bipotent myoepithelial and luminal progenitors have been presented [12,13,14]. A recent study proposed that the origin of BCSC can be traced to luminal progenitors, mature luminal cells or to the bipotent-enriched progenitor cell [15]. This notion was supported by gene signatures from healthy breast cancer cells and BCSC indicating that mutations in a common progenitor or dedifferentiation of mature cells generate BCSC [15]. The mechanism by which stemness can be acquired in a mature cell or progenitor cell however remains to be elucidated.

Expression of proteins involved in cellular reprogramming [16] (including octamer-binding transcription factor 4 (Oct4), sex determining region Y-box 2 (Sox2) and Nanog) has been observed in many different types of cancer, including glioma, pancreatic, lung, prostate and breast cancer [17,18,19,20]. In prostate and lung, Oct4, as well as Nodal, cause an enhanced translocation of β-catenin (for Wnt signaling, see below) to the nucleus, leading to enhanced stemness, and in breast cancer a role for Sox2 in reprogramming of mature cells to BCSC was demonstrated. Sox2, but not Oct4 or Nanog, overexpression was found in breast cancers and nuclear reprogramming of ER-positive Michigan Cancer Foundation-7 (MCF-7) cells yielded Sox2-overexpressing cells with enhanced BCSC characteristics. Sox2 expression was highest in tumors of the luminal B or Her2-positive subtype (~30% Sox2 positive cells), emphasizing a role for Her2 in the governance of stemness in BCSC [18,21]. Interestingly, Sox2 has been shown to be activated by the intracellular domain of Notch [22,23] (for Notch signaling, see below) and together with the observation that Oct4 influences β-catenin nuclear localization, suggest an involvement of dysregulated Notch and Wnt signaling in reprogramming events in BCSC.

## 2. The Notch and Wnt Signaling Pathways

There is a limited number of signaling mechanisms that operate across most metazoan species and are iteratively used to control differentiation of most organs in the body. This “ivy league” group of signaling pathways includes the Notch and Wnt signaling pathways, and they are both important for mammary development and homeostasis as well as contribute to breast cancer when dysregulated. We will here describe the core signaling pathways and then discuss how the Notch and Wnt pathways synergize in signaling.

### 2.1. The Notch Signaling Pathway

**Figure 1:**
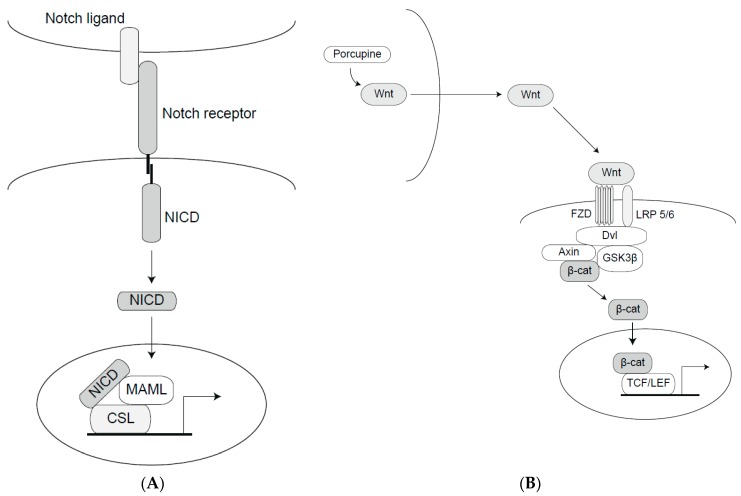
The Notch and Wnt signaling pathways. (**A**) The Notch pathway. A schematic depiction of the proteins in the core Notch signaling pathway. Upon ligand-receptor interaction, the Notch intracellular domain (NICD) is released and forms a ternary complex with MAML and CSL (a DNA-binding protein) in the nucleus to control downstream gene activation. (**B**) The canonical Wnt pathway. The Wnt ligand is modified by porcupine in the ligand-producing cells and interacts with the Frizzled (FZD) receptor. The ligand-activated FZD and Low Density Lipoprotein Receptor-related Protein (LRP5/6) receptors downregulate the activity of the destruction (Dishevelled (Dvl)/Axin/Glycogen synthase kinase 3β (GSK3β)) complex, leading to accumulation of β-catenin (β-cat), and its localization to the cell nucleus. In the nucleus, β-catenin cooperates with T-Cell factor/lymphoid enhancer factor (TCF/LEF) to regulate gene expression.

Notch signaling operates in probably all multicellular organisms, ranging from at least Hydra to humans. It is important for development of most organs in the body and serves in most cellular contexts as a gate-keeper against differentiation, maintaining a stem or progenitor cell state (see [24] for review). The Notch signaling pathway is a cell-cell signaling mechanism where transmembrane ligands and receptors positioned at neighboring cells interact to elicit signaling. Briefly, a Notch ligand (of the Jagged or Delta-like type) on one cell (the signal-sending cell) interacts with a Notch receptor on a juxtaposed cell (the signal-receiving cell) (Figure 1A). Ligand-receptor interaction leads to two consecutive proteolytic processing events in the Notch receptor conducted by ADAMs (a Disintegrin and Metalloprotease) and the γ-secretase complex, respectively, which eventually liberates the C-terminal part of the receptor: the Notch intracellular domain (Notch ICD). Once liberated, the Notch ICD traverses to the cell nucleus, where it interacts with the DNA-binding protein CBF1, Suppressor of Hairless, Lag-1 (CSL) (a.k.a. recombination signal-binding protein-Jkappa (RBP-Jκ) and C promotor-binding factor 1 (CBF1)) and Mastermind-like (MAML) to regulate expression of Notch downstream genes (Figure 1). While the function of the Notch pathway is highly evolutionarily conserved, there are also some recent evolutionary modifications of the pathway. One such example is the presence of two Notch2-related genes in the human genome, which may have played a role in evolution of human-specific brain features [25,26].

We are still relatively ignorant as to why Notch signaling produces so diverse transcriptomic outputs depending on the cell context [24,27]. As discussed below, cross-talk with other signaling mechanisms represents one potential source for downstream diversity. Diversity may, at least in part, also relate to the relatively large number of auxiliary proteins which can in various ways modify and tune the signaling output. These auxiliary proteins range from glycosylating enzymes that modify the extracellular domain of the Notch receptors to a number of enzymes that modify the function and activity of Notch ICD via posttranslational modifications. Thus, the Notch ICD is modified by acetylation, hydroxylation, sumoylation, ubiquitylation and a number of phosphorylations. In the latter category, it has recently been demonstrated that Atypical Protein Kinase C (aPKC) and Proviral integration site for Moloney murine leukemia virus (PIM) kinases phosphorylate Notch ICD [28,29], and the first phosphatase acting on Notch ICD, Eyes absent 1 (Eya1), was recently identified [30]. Several of these modifications affect Notch ICD stability and signaling capacity and are thus interesting candidates from a potential therapy perspective. Another potential source of diversity is that different receptor and ligand paralogs may be endowed with distinct signaling capacities, and the use of a particular ligand-receptor combination may thus produce a specific signaling output. In keeping with this notion, expression patterns of both receptors and ligands are quite complex, and unique receptor-ligand combinations can thus be found in various organs. There is also an emerging notion that all receptors and ligands are not equal in function; specific functions for Delta-like ligand (Dll) Dll1 and Dll4 ligands have thus been observed in vivo [31] and they are also endowed with different signaling dynamics [32]. Moreover, differences in Notch1 and Notch2 receptor function in the kidney as well as in Jagged1 and Dll4 ligand function in angiogenesis have been described [33,34].

### 2.2. The Wnt Signaling Pathway

The Wnt signaling pathway, like Notch, operates in many organs in most multicellular organisms (the name is a combination of the *Wingless* gene from Drosophila and *int* genes from mammals). The Wnt signaling pathway functions both via canonical and non-canonical branches. In the canonical pathway, signaling is mediated via activation of the surface receptors LRP5/6 and Frizzled (FZD) by Wnt ligands, with regulation of the amount of β-catenin as a critical intermediate step (Figure 1B). The amount of β-catenin is regulated via phosphorylation by a destruction complex comprising Glycogen synthase kinase 3β (GSK3β), Casein kinase 1 (CK1), Adenomatous Polyposis Coli (APC) and Axin, and phosphorylated β-catenin is rapidly degraded via proteasomal degradation. Activation by Wnt ligands (there are 19 Wnt ligands in total) leads to stabilization and release of β-catenin from the phosphorylation complex allowing β-catenin to translocate to the nucleus, where it activates gene transcription via interaction with DNA-bound T-Cell Factor/Lymphoid Enhancer Factor (TCF/LEF), and displacing the repressor Groucho (Figure 1B) (reviewed in [35]). More specifically, LRP5/6 phosphorylation upon Wnt-ligand binding sequesters Axin to the cell membrane, which releases β-catenin from the destruction complex. Independently of Axin, the LPRP6 intracellular domain (ICD) prevents β-catenin to be phosphorylated by GSK3β and therefore supports stabilization of β-catenin [36,37]. Axin plays an important role by shuttling between the nucleus and the cytoplasm to relocate β-catenin in the destruction complex. Degradation of Axin via LRP5/6 signaling supports constitutive activation of β-catenin mediated transcription [38].

Non-canonical Wnt signaling comes in two principal flavors, both involving Dishevelled, but not β-catenin. One non-canonical mode operates via intracellular calcium (Ca^2+^) levels and CaMKII (Ca^2+^/calmodulin-dependent protein kinase II), which regulates the transcription factor NFAT (Nuclear factor of activated T cells). An alternative mode involves an interaction between Dishevelled and Ras Homolog Family Member A (RhoA), which in turn regulates Rho Associated Coiled-Coil Containing Protein Kinase (ROCK) and leads to modulation of the cytoskeleton.

## 3. Cross-Talk between Notch and Wnt Signaling

Cells need to integrate complex inputs from the exterior of the cell to produce coordinated, meaningful physiological responses to a complex array of external stimuli. It has been a longstanding discussion how this can be achieved by the rather limited number of “ivy league” signaling pathways, but it is an emerging notion that there exists extensive and complex cross-talks between different signaling pathways to integrate the signaling output and that such cross-talk is both temporally and spatially carefully controlled. The Notch pathway thus interacts with several signaling pathways, including Transforming Growth Factor β/Bone Morphogenetic Protein (TGFβ/BMP), Sonic Hedgehog, and Hippo/Yes Associated Protein/Transcriptional coactivator with PDZ-binding motif (YAP/Taz) [39,40,41,42,43,44,45] as well as with the cellular hypoxic response [46,47,48,49]; for review see [50].

**Figure 2:**
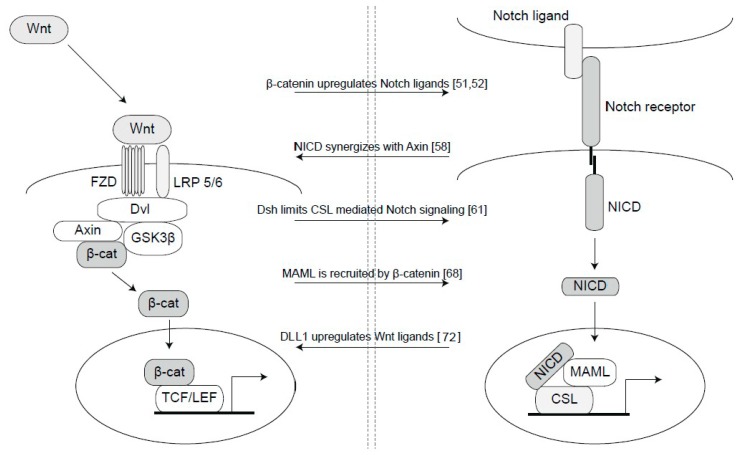
Nodes of interaction between Notch and Wnt signaling. There are a number of interaction nodes between Notch and Wnt signaling, and in the Figure a subset of these are schematically depicted, and the mode of interaction is described in references [51,52,58,61,68,72] (from top to bottom).

Research over a number of years has revealed a rich and complex cross-talk between the Notch and Wnt signaling pathways, and evidence for Wnt-Notch interactions is known from Drosophila to humans, indicating an evolutionarily conservation of intersections between the two pathways (see Figure 2 for depictions of some of the Wnt-Notch interactions). The interactions occur at a number of steps in the two pathways. One mode of intersection is upregulation of Notch ligand or receptor expression by Wnt signaling: upregulation of Jagged1 occurs via β-catenin in hair follicles [51]; the Dll4 ligand is upregulated via β-catenin in the vasculature [52]; and the NOTCH2 receptor is a target of Wnt signaling in colorectal cancer [53]. Interestingly, in a systematic analysis of signaling cross-talk, upregulation of ligands in one pathway by activation of another pathway was found to be a recurrent theme in cross-talk between signaling pathways [54]. The Wnt and Notch pathways synergize also in terms of activation of downstream genes: activation of Mesp2 in somitogenesis requires activation of both Notch and Wnt signaling [55]. In addition, expression of the Notch downstream gene Hes1 is also regulated by β-catenin-mediated Wnt signaling [56]. Another common downstream gene, of particular interest for cancer research, is c-Myc [24,57], and it will be interesting to explore in what contexts Notch and Wnt activates Avian myelocytomatosis virus oncogene cellular homolog (c-Myc) and if the two pathways act synergistically. There are several observations supporting that direct interactions between proteins in the two pathways occurs. A Notch ICD-axin interaction [58] as well as a Notch ICD-Dishevelled and a CSL-Dishevelled interaction have been demonstrated [59,60,61]. Notch trafficking is regulated by binding of Notch to Axin and affected by APC [62]. In Drosophila, the stability of Armadillo, the Drosophila homolog of β-catenin, is controlled by Notch [62,63,64], and a Notch-β-catenin interaction is observed also in mammalian neural progenitor cells [65]. β-catenin has in fact been reported to be part of a ternary β-catenin-Notch ICD-CSL complex, which is located on CSL binding sites in arterial cells [66], and other reports reveal an interaction between GSK3β, β-catenin and MAML [67,68]. The exact binding interfaces for the interaction between β-catenin and components of the Notch transcriptional machinery are not known, but such information would be useful as it may be exploited as a future therapeutic target. GSK3β has, in addition to its role in the Wnt pathway, been shown to serve as a kinase phosphorylating Notch ICD [69,70]. A recent report also indicates a role for GSK3β in Notch1 receptor trafficking [71].

## 4. Notch and Wnt Signaling in Breast Cancer

### 4.1. Notch Signaling in Breast Cancer

Dysregulated Notch signaling, either via direct mutations or via aberrant expression, is linked to a considerable number of cancer forms [24]. Interestingly, Notch signaling is a “goldilocks” pathway [73] meaning that is has to be finely tuned, and mutated forms of Notch can serve as oncogenes or tumor suppressors, depending on the cell- and tissue type. A notable example of tumor suppressor function is the loss of Notch as a cause of skin cancer [74]. In many other forms, gain-of-function mutations or hyperactivated Notch signaling cause tumors. Dysregulated Notch signaling has been linked to breast cancer for well over 20 years, and most of the available data point to an oncogenic function of Notch in breast cancer. In fact, one of the Notch receptor paralogs, Notch4, was discovered via a mouse mammary tumor virus (MMTV) integration into the Notch4 locus, resulting in hyperactivated Notch signaling and breast tumors in mice [75]. Furthermore, reduced expression or loss of the Notch inhibitor Numb is frequently observed in breast cancer [76,77]. Increased expression of NOTCH1 and NOTCH3 has been particularly associated with aggressive basal breast cancer [78,79,80]. Gain-of-function mutations in NOTCH1 and NOTCH2 are found in breast cancer [81], and NOTCH1 translocations are also found in ductal carcinoma in situ (DCIS) [82], which may be considered an early stage in breast cancer development. A recent study extends this analysis, providing support for additional mutations in genes related to Notch signaling in the large data set from the The Cancer Genome Atlas (TCGA) data repository [83]. Furthermore, NOTCH2 and NOTCH3 amplifications are found in basal breast cancers [84,85]. Interestingly, NOTCH3 signaling appears to be constitutive, i.e., ligand-independent, in breast cancer cell lines, which makes it interesting as a therapeutic target [80]. NOTCH3 may also play a role in breast cancer metastasis, as Notch3 expression in breast cancer is important for osteoblast differentiation and TGFβ production [86] as well as for regulation of metastasis to the bone [87]. As regards downstream consequences of Notch activation, activation of c-MYC by Notch appears to be an important event in breast cancer as well as in many other tumor forms [80,88,89,90]. Interleukin-6 (IL-6) is another Notch target gene, although regulated through non-canonical Notch signaling [91]. Quite recently, the ubiquitin ligase Ring Finger protein 8 (RNF8) has been found to regulate Notch1 ICD stability in breast cancer [92] and Notch signaling has been invoked as a control of cellular heterogeneity in TNBC [93].

It is an emerging notion that Notch signaling is linked to BCSC in various ways. Notch signaling has been proposed to drive the genesis and self-renewing potential of BCSC [94,95]. As discussed above, EMT contributes to generating cells with BCSC-like properties [10,96], and it is of note that Notch can contribute to EMT, in particular in conjunction with hypoxia [47], which may contribute to BCSC genesis [10]. Endocrine resistance of BCSCs is controlled by a Notch3/Interleukin 6 (IL6) axis [97]. Conversely, blockage of Notch signaling leads to reduced mammary tumor-sphere-forming capacity as well as a reduced number of BCSCs [98].

Finally, Sox transcription factors represent interesting downstream targets of Notch signaling. Sox2 has been implicated in breast cancer as it is overexpressed in mammary tumors and responsible for BCSC function [18]. Sox2 expression is furthermore a resistance marker for paclitaxel and tamoxifen in breast cancer [99,100]. This may be linked to Notch signaling as Sox2 is activated by Notch1 ICD in TNBC [23], and Notch signaling mediates paclitaxel and tamoxifen resistance in colon and breast cancer cells [101,102]. Sox2 expression levels could thus help to identify drug-resistant BCSC that would respond to Notch inhibition therapy and to define appropriate treatment regiments.

### 4.2. Wnt Signaling in Breast Cancer

An early link between Wnt signaling and breast cancer was demonstrated by an MMTV integration into the Wnt1 locus, which resulted in mammary tumors in mice [103]. Dysregulated Wnt signaling is observed in TNBC, and both the canonical and non-canonical branches of Wnt signaling have been implicated in breast cancer and BCSC control [104] (for review see [105]). A number of mutations in Wnt pathway genes were recently found in the TCGA data set [82], but activating mutations in β-catenin are interestingly not associated with TNBC [106]. Breast cancer patients with elevated Wnt signaling are more prone to develop metastases in lung and brain [107]. BCSCs with high Wnt signaling are more tumorigenic [108], and abrogation of Wnt reduces the metastatic rate [109]. β-catenin can increase expression of EMT genes [110], which is likely to contribute to the ability of dysregulated Wnt signaling to drive breast cancer development. More recently, increased Wnt signaling was suggested to be higher in BCSCs, based on elevated TCF4, LEF1 and β-catenin expression in a subset of Aldefluor-positive BCSCs [109]. In the same report, it was also observed that activation of Wnt signaling led to an increase and conversely, blocking of Wnt to a decrease in the tumor-forming capability of BCSCs [109].

### 4.3. Synergies between Notch and Wnt Signaling Relevant for Breast Cancer

As discussed above, there are ample interaction points between the Notch and Wnt signaling pathways, and it is an emerging notion that Notch-Wnt synergies will be of importance also in breast cancer. One area that may receive particular attention is the interaction between tumor and the surrounding tumor stroma [111]. A recent interesting report describes a Notch-Wnt synergy in a normal mammary setting, demonstrating a complex interplay between Notch and Wnt in the interaction between mammary stem cells and the macrophageal niche [72]. More specifically, expression of the Notch ligand Dll1 on mammary stem cells is important for interactions with macrophages in the stromal niche [72]. In response to Dll1 stimulation, the macrophages express Wnt ligands (Wnt10A, Wnt16 and Wnt3), which in turn are important for mammary stem cell (MaSC) numbers and activity. While studied in normal mouse development, this novel Notch-Wnt axis between mammary stem cells and macrophages would be interesting to study also in a breast cancer context.

Another reason to pay attention to Notch-Wnt interactions is that both pathways are activated by tumor therapy, and this activation contributes to therapy resistance. Thus, both chemo- and radiotherapy lead to enhanced production of Notch and Wnt ligands in the tumor stroma (for review see [112]). Paclitaxel and cisplatin remodel the tumor microenvironment leading to enhanced Jagged1 expression in the tumor stroma, which may elicit increased Notch activation in the tumor proper [113]. Similarly, WNT16B production is enhanced in response to chemotherapy in vivo [114] and doxorubicin treatment increases the expression of different Wnt ligands in a model of TNBC in vitro [115].

A third area is represented by proteins that may exert regulatory control in both the Notch and Wnt pathways, as they may be focal points for therapies that simultaneously affect both pathways. GSK3β represents such a protein and although it has a number of substrates, making it complex as a therapeutic target, it could nevertheless represent an interesting therapy possibility, given that it regulates β-catenin stability as well as acting as a kinase for phosphorylating Notch ICD (see above).

## 5. Notch and Wnt Therapy Development

### 5.1. Notch Therapy Development

While the need for therapies based on Notch modulation is obvious, there are yet no functional Notch therapies routinely used in the clinic. As for all major signaling pathways (which includes Wnt signaling, see below), systemic blockage of the Notch pathway is likely to cause unwanted effects in a number of organs. The failure of a number of clinical trials for γ-secretase inhibitors (GSIs) with the aim of blocking Aβ peptide formation in the brains of patients suffering from Alzheimer’s disease has clearly underlined that a long-term systemic use of agents that completely block Notch signaling gives unacceptable side effects. The off target effects are primarily related to Notch blockade in the immune and gastrointestinal systems as well as in the skin (see [116] for review). With this said, there is however room for improving treatment regiments, and there are a number of strategies aiming at defining tolerable doses of GSI in clinical trials for solid tumors [117]. A recent study reports on dose escalation and dose expansion criteria for use of the GSI LY30309478 for patients with colorectal, ovarian, adenoid cystic carcinoma and breast cancer [118] (for review see [119]). The effect on Notch was not directly measured, but Aβ levels in plasma were significantly reduced, indicating GSI efficiency [118].

It will also be interesting to consider a combination between targeting the Notch pathway and chemotherapy. Data from work in cell lines argue that blocking Notch signaling may make cells more susceptible to chemotherapy. For example, high Notch signaling is linked to therapy resistance in BCSCs via a Jagged1-Notch4 axis [120], and chemotherapy resulted in elevated Notch1 nuclear levels combined with promotion of a BCSC phenotype [121]. BCSCs show higher expression of ABC transporter proteins, which may be caused by hypoxia or EMT, as well as increased DNA repair and anti-apoptotic activities (reviewed in [122]). GSI treatment enhanced sensitivity to doxorubicin in M.D. Anderson Metastasis Breast Cancer (MDA-MB) MDA-MB-231 cells (a basal-type/TNBC cell line) [123]. As Notch signaling controls cellular differentiation in the hematopoietic lineage, attempts to target Notch signaling in tumors may also impact on the immune system. It has been demonstrated that Notch signaling plays a role in orchestrating the tumor environment by increasing the number of monocyte and macrophages in breast [124].

In addition to GSI, which is a pan-Notch inhibition strategy, there are approaches aiming at interfering with specific receptors or ligands, which begin to yield interesting data in pre-clinical systems [125,126,127]. Ligand traps, which have been used successfully in other ligand-receptor systems such as Anaplastic Lymphoma Kinase (Alk) and BMP signaling, is an interesting strategy to modulate Notch ligand-receptor interaction and signaling. This concept has been explored in the vasculature [128,129]. Another potential level for therapeutic intervention is the ternary Notch ICD/CSL/MAML transactivation complex [130,131]. However, blockage at this step of the pathway has to be carefully approached. As a cautionary note, it was surprising to learn that genetic removal of CSL in the basal-like cell line MDA-MB-231, which is expected to abrogate canonical Notch signaling, instead led to more vigorous tumor growth in xenograft experiments in mice [48,132]. Despite these concerns, there is an increasing number of clinical trials that are ongoing or planned for Notch inhibitors, and while most of the trials use GSIs, there is also one trial with a small molecular directed to the Notch transcriptional complex (CB-103) (Table S1).

### 5.2. Wnt Therapy Development

The widespread importance of Wnt signaling in various organs poses the same problem with potential unwanted side effects as for Notch signaling. Currently, there are a number of Wnt inhibitors in clinical development [133]. Wnt can be targeted by porcupine inhibition, as porcupine regulates the attachment of palmitoleic acid to Wnt ligands, which is important for their secretion and transport from the ER [134,135,136]. Interestingly, cells with loss of Notch (head and neck cancer) are very susceptible to Wnt inhibitor (LGK974) treatment [137]. Antibodies and other blocking agents against Wnt ligands or targeting FZD receptors (vantictumab) have been developed and are currently tested in clinical trials [138,139]. Tankyrase inhibitors, which act on PARP (Poly ADP-ribose polymerase) proteins, may also impinge on Wnt signaling, as Tankyrase 1 and 2 are affecting the stability of Axin and other Wnt antagonists [140,141,142]. Intriguingly, a recent study identified Notch receptors as targets of tankyrase mediated degradation, and tankyrases are required for Notch2-mediated signaling [143], pointing to another mode of interaction between the two pathways. A summary of ongoing and planned trials with Wnt inhibitors is found in Table S1.

## 6. Conclusions

To understand cross-talk between leading signaling pathways is a rapidly progressing research area, and to define molecular nodes between pathways is important not only to understand how cells produce appropriate responses to complex external stimuli, but also to understand how dysregulated signaling leads to cancer. In this review, we summarize recent findings from the area of Notch and Wnt signaling, their roles in breast cancer and how the two pathways synergize. Their important roles in breast cancer are becoming increasingly apparent, but there is more work to do before pharmacological targeting of Notch and Wnt signaling become a clinical everyday practice. Advancement in therapy development is rooted in solid understanding of signaling pathway architecture and mechanics, and it is comforting to learn that we have gained new insights in what make these pathways “tick”, and that this information can be useful to identify their Achilles heals for therapy purposes.

## Supplementary Materials

The supplementary materials are available online at http://www.mdpi.com/2227-9059/6/4/101/s1.
